# The Structural and Dynamical Properties of the Hydration of SNase Based on a Molecular Dynamics Simulation

**DOI:** 10.3390/molecules26175403

**Published:** 2021-09-05

**Authors:** Hangxin Liu, Shuqing Xiang, Haomiao Zhu, Li Li

**Affiliations:** 1 National and Local Joint Engineering Research Center of Biomedical Functional Materials, Nanjing 210023, China; 181102031@njnu.edu.cn (H.L.); 201102059@njnu.edu.cn (S.X.); 2 Jiangsu Collaborative Innovation Center of Biomedical Functional Materials, Nanjing 210023, China; 3 Jiangsu Engineering Research Center for Biomedical Function Materials, Nanjing 210023, China; 4 Jiangsu Key Laboratory of Biofunctional Materials, Nanjing 210023, China; 5 School of Chemistry and Materials Science, Nanjing Normal University, No. 1 Wenyuan Road, Nanjing 210023, China

**Keywords:** structural fluctuations, hydration water, protein dynamics transition, mutation, hydrogen bond

## Abstract

The dynamics of protein–water fluctuations are of biological significance. Molecular dynamics simulations were performed in order to explore the hydration dynamics of staphylococcal nuclease (SNase) at different temperatures and mutation levels. A dynamical transition in hydration water (at ~210 K) can trigger larger-amplitude fluctuations of protein. The protein–water hydrogen bonds lost about 40% in the total change from 150 K to 210 K, while the Mean Square Displacement increased by little. The protein was activated when the hydration water in local had a comparable trend in making hydrogen bonds with protein– and other waters. The mutations changed the local chemical properties and the hydration exhibited a biphasic distribution, with two time scales. Hydrogen bonding relaxation governed the local protein fluctuations on the picosecond time scale, with the fastest time (24.9 ps) at the hydrophobic site and slowest time (40.4 ps) in the charged environment. The protein dynamic was related to the water’s translational diffusion via the relaxation of the protein–water’s H-bonding. The structural and dynamical properties of protein–water at the molecular level are fundamental to the physiological and functional mechanisms of SNase.

## 1. Introduction

It is well known that the hydration water of a protein is of great significance to its structure and function; its dynamical properties play a vital role in biochemical processes such as protein folding, molecular recognition, and enzyme function [1,2,3]. Generally, the behavior of water is altered when it is in contact with biomacromolecules [4,5,6,7]. The diffusion of this surface water is slowed about four to seven times relative to the bulk water [8,9,10]. The water residing on the biological molecule surface is called “biological water” [11,12,13,14]. Biological water remains controversial as different experimental techniques have detected different aspects of hydration dynamics. Using NMR spectroscopy, Halle et al. [15] found the affected hydration range was 3–4 Å from the biomacromolecule surface, while Havenith etc. [16] inferred that the thickness of the hydrated shell was ~20 Å from their terahertz spectroscopy measurement, indicating the profound impact of the biomacromolecules on the hydration water.

The polar, or ionic, groups of biomolecules interact with water molecules through hydrogen bonding and coulomb forces [17,18], forming a coupled state with unique dynamic characteristics, which have multiple levels of complexity. Water–protein coupling can be described by the characteristics and dynamics of hydration, which mainly include the translational, rotational, and hydrogen bonding actions of water molecules near protein surface. To explore this coupling state, many efforts have been devoted to the exploration of the dynamical properties of various hydration proteins and water through different experimental techniques, including neutron scattering, NMR spectroscopy, depolarized light scattering, dielectric spectroscopy, terahertz spectroscopy, 2D-infrared spectroscopy, etc. [19,20,21,22,23,24,25,26]. Doster et al. [27] found that the Mean Square Displacement (MSD) of myoglobin hydrated in water showed a sudden increase at around 180 K. This phenomenon became known as Protein Dynamic Transition (PDT) and was considered as a microscopic feature of protein bioactivity. Since then, PDT has been studied in a variety of hydrated proteins and in different solvation environments [28,29,30]. Tarek et al. [31,32] deduced that this behavior originated in protein–water–hydrogen bonding. Other studies highlighted the vital role of heterogeneous local charge distribution on protein surfaces in ruling hydrogen bonds with water as well as coupling [33,34].

In many studies, staphylococcal nuclease (SNase) was observed to be an important model system. SNase is a Ca^2+^-dependent endo-exonuclease of 149 amino acids and is usually used for the non-specific cleavage of DNA or RNA substrates in biology. With only single tryptophan residue (W140), SNase is intrinsically an ideal model for transient fluorescence studies. Using site-directed mutagenesis, Zewail et al. [35] replaced several charged changed residues around W140 with hydrophobic alanine ones and measured their Stokes shifts and solvation dynamics. They believed that a long-time scale of hydration decrease arises through fluctuations at the protein’s surface. Similar work was also carried out by Qin et al. [34], with some differences in mutations. They checked the fluorescence transients with temperature changes and found that the charge mutation changed the relaxation dynamics of hydrated water, and that the side chain relaxation was strongly coupled with hydration water. Focusing on the hydrogen exchange behavior of SNase, Skinner et al. [36,37] analyzed in detail the relationship between the exchange competence and the wide-range dynamic excursions of protein through NMR spectroscopy studies. Molecular dynamics simulations of SNase and its mutations showed the diverse pattern of hydration of internal cavities, which could explain the different crystal structures observed under cryogenic and room temperature conditions [38]. Through MD simulations, the ionization state and orientation of Glu-Lys pairs buried in SNase were found to be related to the hydration level of the cavity, which suggested a general understanding of the protein stabilizing mechanism [39]. SNase is known to have a partner-induced folding phenomenon [40], which ensures that its highly disordered structure of can fold properly and regain enzymatic activity under physiological conditions when in contact with its partner molecules. Revealing this mechanism of coupled folding and binding largely depends on a good understanding of the way protein interacts with water.

In this study, molecular dynamics simulations were applied to characterize the structural and dynamical properties of SNase and the associated hydration water at different temperatures. The mutations at different levels were also taken into consideration [41]. The Protein Dynamic Transition(PDT) was analyzed based on MSD calculations. The residence and reorientation dynamics of superficial water as well as hydrogen-bonding relaxation methods were studied and are discussed in this paper. This study provides molecular-level information for understanding the protein–water coupling state and may lay the foundation for subsequent studies of SNase recognition with DNA and drug development.

## 2. Results and Discussion

### 2.1. Structural Fluctuations

**Figure 1:**
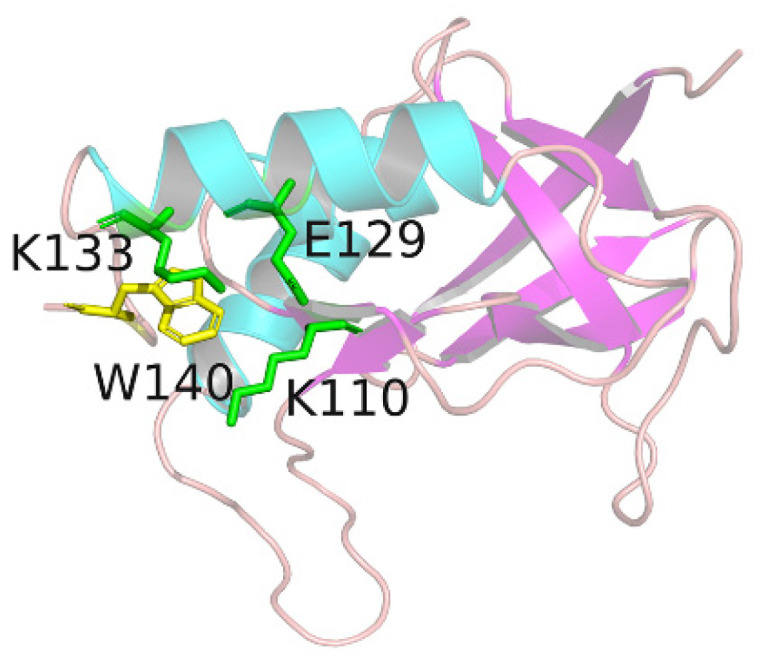
The 3D structure of SNase. W140 is shown in yellow, and the surrounding K110, E129, K133 are shown in green.

As performed in an experiment by Qin [34], three residues, lysine(K110), glutamate(E129), and lysine(K133), spatially located around the tryptophan(W140), were successively mutated to alanine. As shown in Figure 1, SN0 represented the original SNase without mutation, SN1 corresponded to the K110 mutation, SN2 stood for the double mutation of K110 and E129, and SN3 for the three residues were all mutated to alanine. All mutations are shown as 3D structures in Figure S1 (Supplementary Materials).

**Figure 2:**
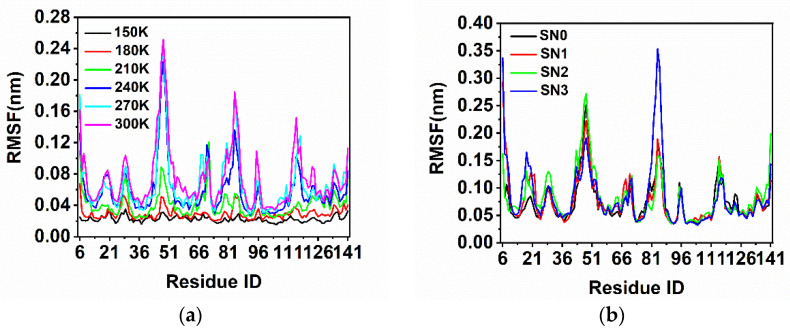
The changes in RMSF of residues in each system: (**a**) changes in RMSF of SN0 at different temperatures; (**b**) changes in RMSF under different mutation conditions at 300 K.

The Root Mean Square Fluctuation (RMSF) represented the flexibility of each residue on SNase by measuring the average position fluctuations. RMSF was calculated to describe the fluctuation of atomic positions and the flexibility of various regions of SNase. Figure 2 shows that the atomic fluctuations were limited, with an RMSF of 0.2–0.3 nm at low temperature (below 210 K). Hoever, from 210 K to 300 K, the fluctuation remarkably increased, corresponding to the greater flexibility of SNase. At 300 K, the overall amplitude of fluctuations was more pronounced, especially K84, which had a large amplification after triple mutations (as SN3). Those residues that display relatively intense fluctuations are not enclosed inside the protein but spread out on the surface; they are sensitive to the external movement of the surrounding water molecules. In Figure S2 a cartoon display is used in order to show the positions of these residues on the protein (Supplementary Materials).

The effects of mutations on the protein structure were mainly analyzed through monitoring the evolution of the protein’s secondary structure (Supplementary Materials, Figures S3 and S4). The statistics relating to the structural components are listed in Table 1. As can be seen, except for the loss of some β-sheet structures and the formation of a coil structure in SN3, the rest of the structural differences were not obvious. The results showed that the mutations had little effect on the structure of protein, based on 20 ns MD simulations. The stable and near-identical structures of the protein backbones made the subsequent hydration dynamics analyses comparable among mutations. Although no effects of these mutations on the SNase structure were found in other experiments either [33], it should be noted that the complete conformational evolution of this protein relies on a much longer MD simulation time than the current 20 ns, and the final conclusion about structural changes can only be drawn by sufficient sampling.

**Figure 3:**
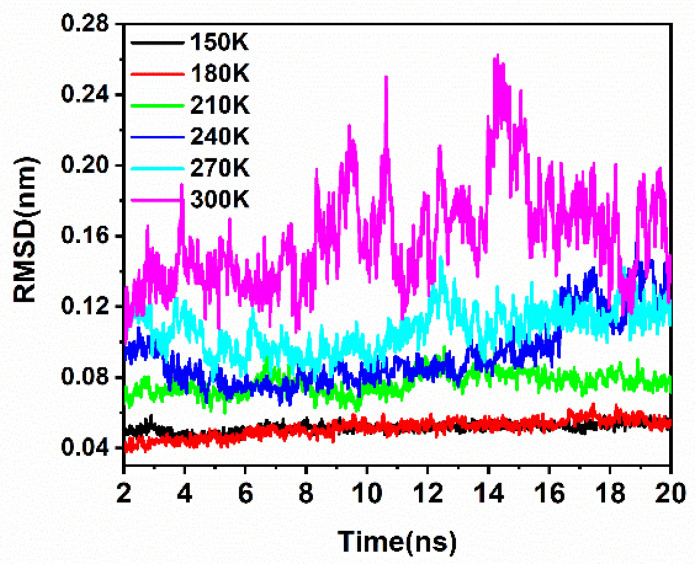
The RMSD of C_α_ in SN0 at different temperatures during 20 ns simulations.

The solvent accessible surface area (SASA) is an estimated surface area of protein atoms. Because of its potential for contact with solvent molecules, it is also used as an indicator of protein conformation changes with the surrounding medium [42,43]. As Table 2 shows, the SASA of SN0 became larger with the raising of the temperature, suggesting the looser conformation the protein might adopt at high temperatures. This means that the higher the temperature, the more intense the structural fluctuation is, resulting in a higher degree of exposure to water. The Root Mean Square Deviation (RMSD) was used to monitor the time-dependent changes to the structure. Here, the RMSD reflected the mobility of the SNase atoms, providing an observational perspective on the dynamics of SNase. In Figure 3, the RMSD of SN0 C_α_ increased in line with the temperature, especially above 210 K, which was consistent with the results of the RMSF.

### 2.2. Radial Distributions Function

**Figure 4:**
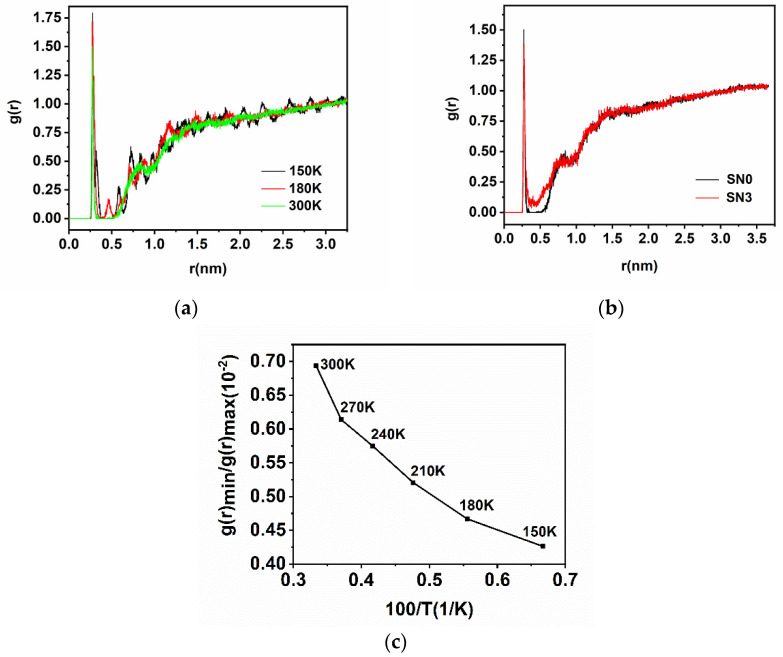
The nitrogen atom on W140 was centered and water oxygen atoms were calculated for RDF in different conditions: (**a**) the RDF of SN0 at 150 K, 180 K and 300 K. (**b**) the RDF of native SN0 and mutation SN3 at 300 K. (**c**) the relationship between the ratio of g(r)min/g(r)max to SN0 and temperature. Parts of the data (the native at 210 K, 240 K, 270 K and mutations SN1, SN2 at 300 K) are not plotted to avoid overlap among curves.

The structure of water molecules at the protein interface can be described by radial distribution function (RDF), and the calculated distance of the first minimum in RDF can be considered as the thickness of the hydration layer around a given location [44]. For the present study, a nitrogen atom on W140 was centered and water oxygen atoms were calculated for RDF. Figure 4a features the RDF of SN0 at different temperatures. Figure 4b shows the RDF of the wild SN0 and the triple mutation SN3 at 300 K. The height and position of the first peak in all systems are listed in Table 3. Water were molecules arranged in an orderly manner at low temperature, especially at 150 K. As far as 3 nm away from the location of concern, these water molecules remained coordinated and sterically restricted, demonstrating a long-distance influence on the solvation–water structure from the protein. This ordered structure gradually disappeared as the temperature rose. Two hydration layers could be roughly distinguished at 300 K, with the first layer extending to a distance of ~0.35 nm and the second to ~1.0 nm. The height of the first maxima in the mutation systems continuously decreased, revealing that the ordered arrangement of W140 hydration was faded by the less charged environment as interactions between water molecules and ionic residues (lysine or glutamate) were absent upon mutations.

The ratio of the first non-zero minima to the first maxima (g(r)min/g(r)max) (Figure 4c) was calculated to represent the de-packing of the first hydration layer in the structure [45]. The ratio increased approximately linearly in the range of 150 K to 270 K, which indicated the tendency of the first hydration layer to gradually lose its structure as the temperature increased, and of the water molecules to swap between the first maxima and minima regions. From 270 K to 300 K, there was a relatively large increase, departing from the linear rate, illustrating that the water molecules in the first layer were less coordinated and the boundary of hydration shell became indistinct.

### 2.3. Mean Square Displacement of SNase and Hydration Water

**Figure 5:**
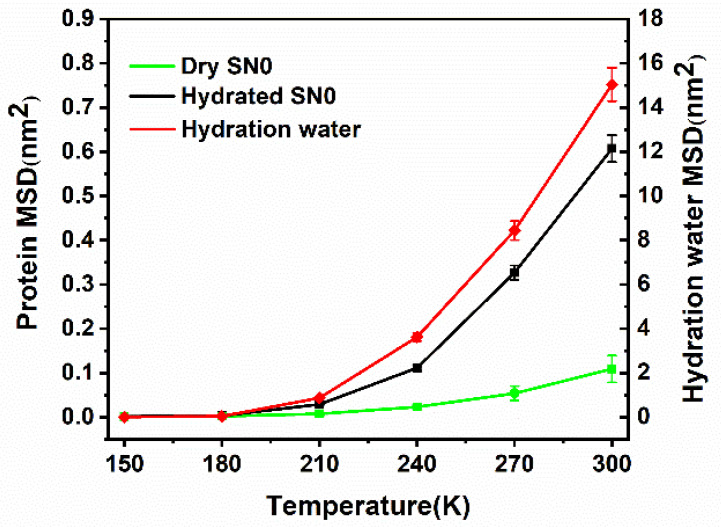
MSD versus temperature. The hydrated SNase is shown in black, the dry SNase in vacuum is shown in green, and the hydration water near the protein is in red. The hydration shell was defined as the water molecules within 0.6 nm of the protein. The MSD was calculated for protein no-H atoms. The 19 ns trajectory was divided into 95 periods. The MSD difference in each period (200 ps) was obtained and the final value was averaged over all periods with standard deviation [45].

The Mean Square Displacement (MSD) of the SNase and hydration water were calculated to verify the typical characteristics observed in experiment. The details of the MSD calculation are shown in Section 3.2.3. Figure 5 shows the statistical average of the MSD at different temperatures using a multiple-time origin method. The results showed that the MSD increased slightly with temperature (150 K to 210 K) for both SNase and hydration water in an approximately linear manner. As the temperature rose (>210 K), the MSD increases of both protein and hydration water were pronounced in their deviation from the original trend. The result showed the typical experimental trend of protein dynamic transition (PDT). It is widely believed that this dynamical transition is closely related to protein function. Tarek et al. [31,32] deduced that this phenomenon originates in protein–water–hydrogen bonding. Roh suggested that the non-linear change in the MSD of myoglobin was caused by the excitation of non-vibrational motions [46]. In the present study, this variation of the MSD with temperature could be regarded as a transition from a low-diffusion state to a relatively high-diffusion state. The simultaneous changes in the MSD of hydrated protein and hydration water confirmed the existence of a protein–water coupling state. In the absence of water, the relatively small degree of protein thermal motion revealed that the coupling state had peculiar dynamical characteristics.

### 2.4. Hydration Dynamics Based on Mutation

**Figure 6:**
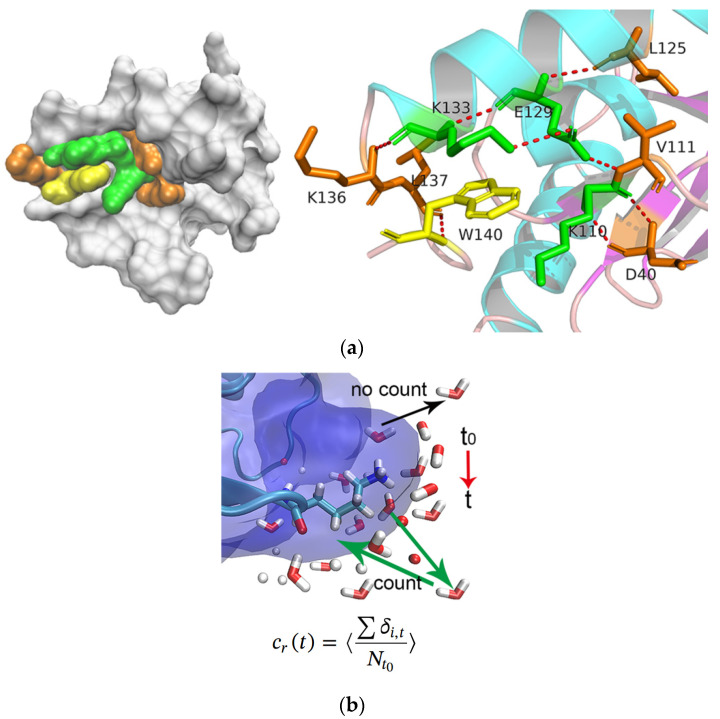
Analysis of residence dynamics: (**a**) the position of the selected region in protein including W140 and the three mutant residues as well as several other residues nearby, D40, V111, L125, K136, and L137; (**b**) the calculation of *C_r_*(*t*) and counting rules, where *N_t0_* is the water molecules number in the first coordination shell at t_0_, $\delta_{i,t}$ equals to 1 if the ith water is in the shell at time t else 0, and the angle bracket denotes the ensemble average.

In this study, the residence and rotation correlation functions of water molecules sur-rounding the mutation sites were calculated to suggest the hydration dynamics. As Figure 6a shows, W140 and the three mutant residues, as well as several other residues nearby, D40, V111, L125, K136, and L137, were taken as the reference positions on the protein’s surface, and the water molecules within 0.6 nm of protein were selected as targets for analysis. The calculation of the residence correlation curve *C_r_*(*t*) and the counting rule are shown in Figure 6b. The rotation correlation functions of the water molecules surrounding the mutation sites were calculated to characterize the reorientation dynamics.

The *C_r_*(*t*) curves are displayed in Figure S5 (Supplementary Materials). To gain more insight into this issue, the curves were further fitted by a stretched exponential function, which can accurately describe the relaxation of water *C_r_*(*t*) in amorphous and disordered systems like protein surfaces [47,48]. The fitting function is given in Section 3.2.5. The results are listied in Table 4. The residence times exhibited a biphasic distribution, with two time scales, 4.8–3.1 ps and 58.9–46.3 ps, revealing the different ways in which water escaped from the hydration space. The fast process corresponded to the water–water exchange at the boundary between the hydration space and the outside. Water molecules at the boundary far from the protein surface were less affected by the protein than those in close contact with it, so the exchange process was relatively fast. The slow process was related to the entire diffusion behavior of the hydration water under the influence exerted by charged groups on the protein, representing the protein–water collective motion.

The hydrated water network on the protein surface relaxed and coupled to the motion of local protein on picoseconds. For mutations, the absence of charged groups and the minimized steric hindrance brought about the alleviated binding effect of the protein surface on local water. More free movement of water molecules made the process of diffusion faster relative to those without mutation. The acceleration illustrated the impact of chemical heterogeneity and the topological character of protein surfaces on hydration dynamics.

The reorientation dynamics of water molecules in hydration layer can be mediated by the electrostatic interaction between the water dipole and charged groups on the protein surface. The dipole autocorrelation function of the water molecules was calculated for analysis of this issue (Section 3.2.5) and the *C_μ_*(*t*) curves are shown in Figure S6 (Supplementary Materials). The characteristic orientation retention time t*_μ_* of hydration water was obtained by fitting *C_μ_*(*t*) with a single exponential function (Section 3.2.5). The results are listed in Table 5. By comparison, the SN0 without any mutation displayed the longest orientation reservation time, 4.8 ps, and the gradually shortened t*_μ_* in mutations unambiguously confirmed that the charged group of proteins could delay the orientation motion of the surrounding water. Besides, the local morphology of the protein surface was another possible factor responsible for this trend, as the residues containing relatively large sidechains had been replaced with alanine in the mutation. The smaller sidechain meant that more free space was left for the water motions. The bulk water molecules dynamically adjusted their position and direction to adapt to the dynamic reconstruction of the hydrogen bonding networks in which they participated. The retarded rotation of hydration water was also an indication of the hindered hydrogen bonding dynamics in the charges’ crowded environment.

### 2.5. Hydrogen Bonding Dynamics

Hydrogen bonding contributes greatly to the structure and dynamics of protein hydration. In this work, geometric criteria were adopted to define the formation of a hydrogen bond [49]. If the distance between the hydrogen bond donor (D) and the acceptor (A) was less than 3.5 Å, and the hydrogen atom-D-A angle was less than 30°, an H-bond formation would be registered. The selected region is shown in Figure 6.

**Figure 7:**
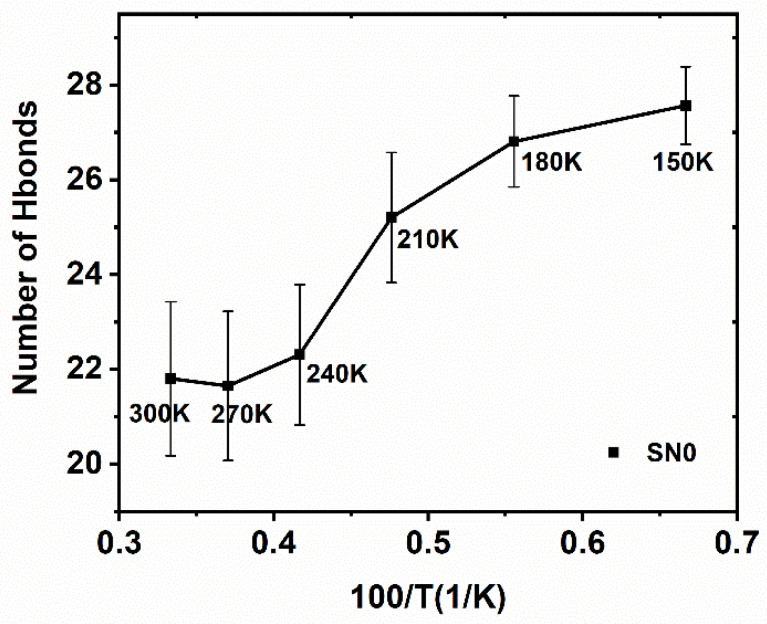
The relationship between the temperature and number of hydrogen bonds formed by water and the selected region of protein. Statistics on 1900 frames with standard deviation.

As shown in Figure 7, the number of hydrogen bonds thended to decrease in a non-linear manner as the temperature rose. From the perspective of the MSD, the protein seemed frozen at 150–180 K and formed relatively more H-bonds with water. The low temperature lead to the low motility of the water, which made the H-bond formation enthalpy occupy a major share of the energy landscape. From 150 K to 210 K the MSD increased by little, while the H-bond lost about 40% in the total change, mainly because the protein–water H-bond formation enthalpy was partly neutralized by a favorable entropy increase derived from the enhanced dynamics of the water. However, this could still not totally activate the protein. Above 210 K, the average energy barrier to H-bonds exchanging, mostly between water and water, gradually fell into the range of thermal fluctuation (Kb) and those formerly established protein–water H-bonds became unstable. Hydration water molecules have comparable capabilities and opportunities to make H-bonds with protein and with the other water molecules, resulting in the final activation of the protein’s thermal movement.

**Figure 8:**
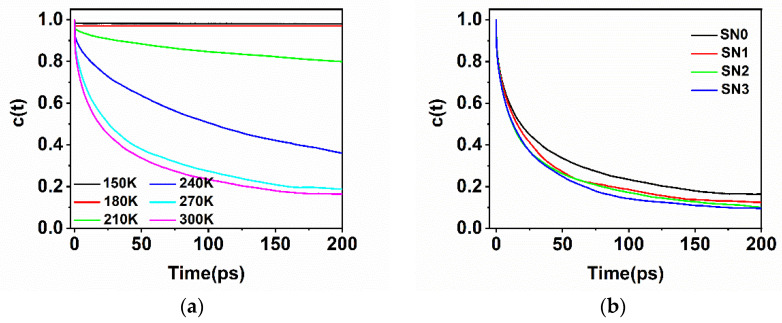

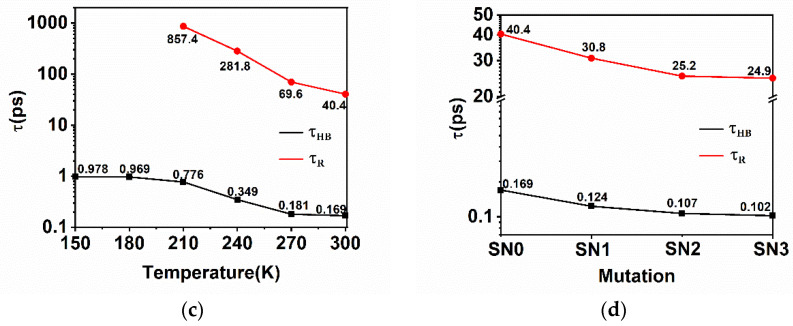
Hydrogen bonds between water and selected regions of the protein: (**a**) hydrogen bond existence autocorrelation function of SN0 at different temperature (**b**) hydrogen bond existence autocorrelation function of different mutations (**c**) hydrogen bond lifetimes τ_HB_ (in black) and relaxation times τ_R_ (in red) of SN0 at different temperatures. (**d**) hydrogen bond lifetimes τ_HB_ (in black) and relaxation times τ_R_ (in red) of different mutations. The calculation methods of the τ_HB_ and the τ_R_ can be seen in the Materials and Methods section.

As Figure 8 shows, the rapid decay (τ_HB_) on the time scale of hundreds of femtoseconds related to the process of the breaking of hydrogen bonds was caused by the librational (hindered rotational) motions of water molecules. This process reflected the exchange of hydrogen bond partners; when the water hydroxyl (OH) trades the H-bond acceptor, it triggers the reorientation of water molecules through a sudden large angle jump [50]. The c(t) also showed a secondary decay on the picosecond time scale, reflecting the relaxation of the protein–water hydrogen bond that resulted from multiple factors, including diffusion, hydrogen bond exchange, and the intrinsic properties of protein. The τ_HB_ was basically at the same level in low temperature ranges, 0.978, 0.969, 0.776 ps, and was largely shortened to 0.349, 0.181, 0.169 ps at high temperatures, indicating that the rapid H-bond relaxation originated from the nature of aqueous media and was subjected to the dynamics of the protein. As Figure 8a shows, the black line of temperature point 150 K and the red of 180 K were basically kept flat. The slow decay was absent in c(t) at low temperatures, which was related to the freeze state mentioned above. The protein–water was nearly steadily H-bonded, with a long relaxation time far beyond the observation time. Above 210 K, the slow relaxation time was obviously shortened from 857.4 ps at 210 K to 40.4 ps at 300 K, meaning that the slow relaxation time was highly correlated with the dynamics of the protein.

**Figure 9:**
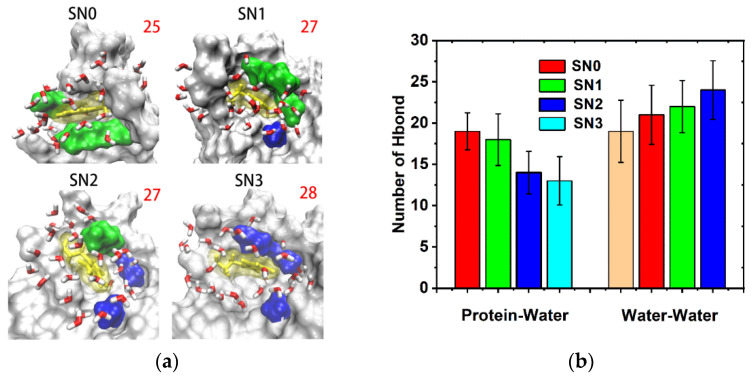
Changes in the number of water molecules and hydrogen bonds around the mutation: (**a**) the number of water molecules within 0.5 nm around W140. W140 is shown in yellow, non-mutated residues are shown in green, and mutated residues are shown in blue, while the number of water molecules is marked in red for each mutation; (**b**) the average number of hydrogen bonds formed by protein and water molecules close to W140, and that of water molecules with themselves. Statistics on 1900 frames with standard deviation.

The aggregation of the protein with the other molecules was actually achieved by hydrophobic effects, electrostatic interactions, or hydrogen bonding. The arrangement of the water molecules around W140 and the local hydrogen bonding pattern played a vital role in their activity and recognition ability. The number of water molecules within 0.5 nm around W140 is shown for all mutations in Figure 9a. As a result, they changed little. 

However, the mutations did change the arrangement of hydrogen bonding. As shown in Figure 9b, these waters made fewer H-bonds with protein in the more hydrophobic environment, and established more H-bonds with themselves. The charged groups intensively interacted with water molecules by constraining them in translation and rotation, making it relatively difficult for the water molecules to form H-bonds with other water molecules. By replacing the charged sidechains with methyl groups, the original restriction on the water molecules disappeared, making the water molecules freely participate in the formation of water–water hydrogen bonds.

The electrostatic attraction between protein and water also had a stabilization effect on the H-bonding. With the mutation from the charged residue to alanine, local electrostatic attractions were weakened, resulting in a shortened lifetime, τ_HB_, from 0.168 ps in SN0 to 0.102 ps in SN3. Given that the amount of hydration water was basically identical in all mutation systems, the accelerated rupture of protein–water hydrogen bonds could be attributed to the rapid rotation of the water, which lacked the stabilization effect of the more hydrophobic environment. As described by τ_R_ in Figure 8, the water molecules lost their opportunity to preserve the hydrogen bond they previously made with the protein; the translational motion of the water molecules mainly accounts for the slow dynamics. The τ_R_ tended to became smaller with mutations, from SN0 (τ_R_ = 40.4 ps) to SN3 (τ_R_ = 24.9 ps), because the diffusion of the water molecules was less affected by the electrostatic attractions or steric hindrances in the hydrophobic environment than in the crowded environment of the charged groups. The enhanced water–water interactions made the hydration water take part easily in the hydrogen bonding network with other water molecules and escape from the original hydrogen-bonding “site”.

## 3. Materials and Methods

### 3.1. Molecule Dynamics Simulation

**Figure 10:**
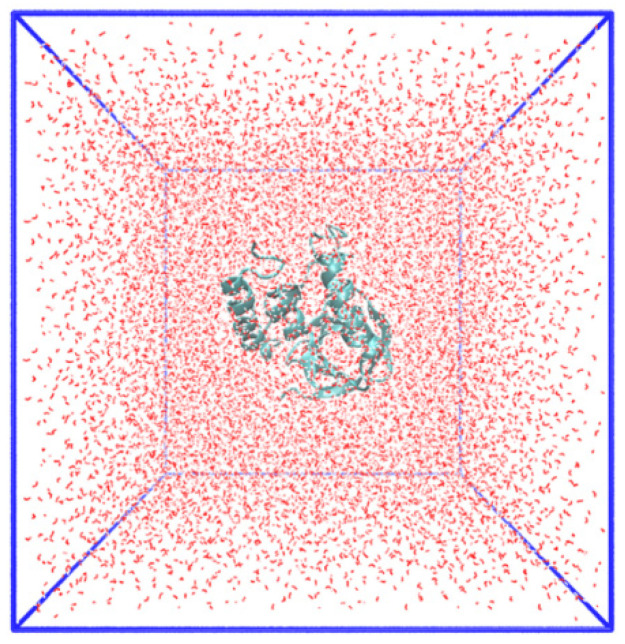
Simulation system protein centered in the 7 nm × 7 nm × 7 nm box.

The SNase structure was selected from the protein database (ID:1SNO). Six temperature points of 150 K, 180 K, 210 K, 240 K, 270 K, and 300 K were set for SN0 in order to study the PDT phenomenon. Three mutation systems were simulated in constant 300 K. In each simulation system, protein was centered in a box of 7 nm × 7 nm × 7 nm with a periodic boundary (Figure 10). An OPLS-AA/L [51,52] force field was used to describe the protein and a SPC/E [53] model was used for the water molecules; their good performances were in line with their performances in other experiments [54]. The temperature and pressure were controlled by the Berendsen method [55]. The pressure was 1.01 × 10^5^ Pa with a pressure coupling constant of 2.0 ps. A LINCS algorithm was set in order to constrain the intramolecular bonding interaction [56]. The non-bonding van der Waals interaction was calculated using L-J potential with a 1.0 nm cutoff distance. A particle-mesh Ewald method was used to deal with the electrostatic interaction [57]. Energy minimization was first performed used the steepest descent method to remove improper placements. Subsequently, a 2 ns NVT run and a 2 ns NPT run were sequentially carried out for equilibrium. Finally, a 20 ns MD run with a 2 fs step size was performed in an NPT ensemble for each system, and the last 19 ns dates were collected for subsequent analysis with a saving frequency of 10 ps. Additionally, a 3 ns MD run was performed with a 1 ps saving frequency for residence dynamics analysis; a 1 ns MD run with a 2 fs saving frequency was used for the analysis of hydration bonding dynamics and a 50 ps MD run with a 1 fs saving frequency was used for the analysis of rapid reorientation dynamics. All MD simulations were performed using GROMACS 5.05 [58]. As an advanced version, the OPLS-AA/M force field (not used in the current work) would have been expected to offer a better performance [59].

### 3.2. Analysis Methods

#### 3.2.1. Root Mean Square Fluctuation

The Root Mean Square Fluctuation (RMSF) was calculated by the following equation:

(1) $$RMSF=\sqrt{\sum_{t=1}^{T}{\left(x{\left(t\right)}_{i}-\bar{x_{i}}\right)}^{2}},$$

where $x{\left(t\right)}_{i}$ refers to the coordinates of the particle *i* at moment *t* and $\bar{x_{i}}$ is the mean coordinate of position.

#### 3.2.2. Root Mean Square Deviation

The Root Mean Square Deviation (RMSD) was calculated by:

(2) $$RMSD=\sqrt{\frac{1}{n}\overset{N}{\underset{i=1}{\sum }}{\left|v_{i}-w_{i}\right|}^{2}}$$

where$\;v_{i}$ and $w_{i}$ represent the two groups of C_α_ sites, respectively. In this study, the referential group was C_α_, set at a previous time.

#### 3.2.3. Mean Square Displacement

The solvent mobility is described by the diffusion coefficient related to the slope of the molecule’s Mean Square Displacement (MSD). It can be expressed by:

(3) $$MSD={\langle |r_{i}\left(t\right)-r_{i}\left(0\right)|}^{2}\rangle$$

where $r_{i}\left(t\right)$ and $r_{i}\left(0\right)\;$are the position vectors of the molecule i at the time *t* and *t*_0_.

#### 3.2.4. Radial Distributions Function

The radial Distributions Function (RDF) can be expressed by this equation:

(4) $$g_{AB}\left(r\right)=\frac{\langle \rho_{B}\left(r\right)\rangle }{\langle \rho_{B}\rangle {}_{local}}=\frac{1}{\langle \rho_{B}\rangle {}_{local}}\frac{1}{N_{A}}\overset{N_{A}}{\underset{i\in A}{\sum }}\overset{N_{B}}{\underset{i\in B}{\sum }}\frac{\delta \left(\left|r_{i}-r_{j}\right|-r\right)}{4\pi r^{2}}$$

where$\;\langle \rho_{B}\left(r\right)\rangle$ is the density of the water at a distance r from the protein, $\langle \rho_{B}\rangle {}_{local}$ is the density of the bulk water, and $\left|r_{i}-r_{j}\right|$ is the distance between the protein atom and the water oxygen.

#### 3.2.5. Hydration Dynamics Analysis Based on Mutation

The number of water molecules in multiple groups of 200 ps time periods was coefficient-averaged and normalized for the analysis of residence dynamics. The residence dynamics curves are further fitted by stretched exponential function as follows:

(5) $$C_{r}\left(t\right)=n^{s}e^{-{\left(\frac{t}{\tau_{s}}\right)}^{\gamma }}+n_{1}e^{-\frac{t}{\tau_{1}}}$$

*τ*_1_ and *τ^s^* correspond to the fast and slow decay in residence dynamics, *n*_1_ and *n^s^* are the corresponding attenuation coefficient, and *γ* is an index for the evaluation of the structural order of the solute.

The reorientation autocorrelation function is defined as follows:

(6) $$C_{\mu }\left(t\right)=\langle \vec{\mu \left(0\right)}\cdot \vec{\mu \left(t\right)}\rangle =\langle \cos\xi \left(t\right)\rangle \;$$

where μ(0) and μ(t) are the dipole vectors of the water molecules at moments *t*_0_ and *t*, respectively, and the angle bracket represents the ensemble average. The fitting exponential function is as follows:

(7) $$\;C_{\mu }\left(t\right)=A_{\mu }e^{-\frac{t}{t_{\mu }}}\;$$

where $t_{\mu }$ is the characteristic reorientation time, and $A_{\mu }$ is the preexponential factor.

#### 3.2.6. Hydration Bonding

The fast hydrogen bond lifetime, τ_HB_, is defined as the average time that a given protein–water hydrogen bond remains intact, which corresponds to rapid hydrogen bond formation and breaking. The relaxation time of the slow hydrogen bond is defined by the attenuation of the bond correlation function c(t) = <h(0)h(t)>/<h> [60]. It is a hydrogen-bond population operator. It equals to 1 if a given pair is hydrogen-bonded time, t, otherwise it equals to 0. The angle brackets indicate the ensemble average. c(t) is the probability that a random D-A pair is hydrogen-bonded at time t = 0 and still holds the bond at time t, regardless of whether the bond breaks at an intermediate time. The relaxation time, τ_R_, of the hydrogen bond is defined as the decay of c(t) to 1/e [60].

## 4. Conclusions

This work presented clear proof of the coupled state between SNase and hydration water. As the temperature rose, a nonlinearly increase in the Mean Square Displacement (MSD) of the protein was found around 210 K, which was synchronous with that of the hydration water. From 150 K to 210 K, the MSD of the protein increased by little, while the protein–water hydrogen bonds lost about 40% in the total change. The protein motion was highly enhanced above 210 K; meanwhile, the hydration water demonstrated a comparable trend in making H-bonds with protein– and other waters. These observations highlight the importance of hydrogen bonding in the hydration dynamics of protein. By characterizing both the fast and slow protein–water H-bond dynamics, it was found that the rapid H-bond lifetime (<1 ps) was related to the rotational movement of the water molecules, and that the slow characteristic relaxation time (from 857 ps at 210 K to 40 ps at 300 K) was due to the diffusion behavior. It can be inferred that the protein’s dynamical transition is connected to the water’s translational diffusion via the relaxation of protein–water H-bonding. Three local charged residues were mutated into alanine. It was shown that the characteristic residence time of the hydration water fell from 58.9 ps to 46.3 ps, as the local chemical environment became hydrophobic due to the mutations. The reorientation dynamics of the water molecules also became faster, with the characteristic reorientation time decreasing from 4.8 ps to 3.8 ps; protein–water H-bonding relaxation had the slowest lifetime, 40.4 ps, for the wild-typed, and the fastest, 24.9 ps, for the triple mutations. It could be suggested that the recognition ability of biomolecules in physiological environments and the specificity of the individual protein, in its chemical composition and molecular structure, determine the unique protein–water coupling state. In dynamical terms, the distinguishing features of the protein–water collective motion generate extensive information. This work could be helpful to the understanding of protein–water interactions and how such interactions drive protein function at the molecular level. It also provides an important perspective, the hydration effect, that can be applied to the study of biological recognition as well as activity.

## Figures and Tables

**Table 1:** Secondary structure statistics of mutation systems.

Mutation	SN0	SN1	SN2	SN3
Coil(%)	17	19	18	20
β-Sheet(%)	28	27	28	26
β-Bridge(%)	2	3	2	2
Bend(%)	14	11	12	12
Turn(%)	12	14	13	14
α-Helix(%)	25	24	25	25
3-Helix(%)	2	2	1	1

**Table 2:** SASA of SN0 at different temperatures; statistics on 1900 frames with standard deviation.

Temperature (K)	150	180	210	240	270	300
**SASA (nm^2^)**	80.2 ± 0.6	81.5 ± 1.1	82.9 ± 0.8	83.9 ± 1.3	84.4 ± 1.2	85.6 ± 1.6

**Table 3:** The value of the first peak of RDF in each system.

Mutation	SN0						SN1	SN2	SN3
Temperature (K)	150	180	210	240	270	300	300	300	300
Position (nm)	0.276	0.274	0.274	0.272	0.27	0.272	0.272	0.276	0.276
Height	1.794	1.719	1.667	1.554	1.513	1.503	1.491	1.415	1.381

**Table 4:** Fitting results for *C_r_*(*t*) in mutations. The uncertainty is the mean square deviation between the raw data and the best fitting value.

Mutation	SN0	SN1	SN2	SN3
τ^s^ (ps)	58.9 ± 0.35	57.8 ± 0.30	49.4 ± 0.40	46.3 ± 0.36
τ_1_ (ps)	4.8 ± 0.05	4.7 ± 0.05	4.3 ± 0.05	3.1 ± 0.13

**Table 5:** Fitting results for *C_μ_*(*t*) in mutations. The uncertainty is mean square deviation between the raw data and the best fitting value.

Mutation	SN0	SN1	SN2	SN3
t*_μ_* (ps)	4.8 ± 0.01	4.3 ± 0.01	4.2 ± 0.01	3.8 ± 0.00

## Data Availability

Data are contained within the article or supplementary material.

## Supplementary Materials

The following are available online. Figure S1: The local structures of the mutation system, named SN0, SN1, SN2, SN3. The unmutated structures are shown in blue and the mutated structures are shown in red. Figure S2: The position of the residues with large changes in RMSF in the protein structure (SN0, SN1, SN2, SN3). Figure S3: Secondary structures of the mutation systems during the 20 ns. Figure S4: The residence time of water within 0.6 nm of the selected area in the mutation systems. Figure S5: Autocorrelation curve of the hydration layer dipole fate of each mutation system, Figure S6: The reorientation dynamics of hydration water in mutation systems.

## Sample Availability

Samples are not available from the authors.
