# Supplementary material for: The Structural and Dynamical Properties of the Hydration of SNase Based on a Molecular Dynamics Simulation
Source: Molecules. 2021 Sep 5;26(17):5403. doi: 10.3390/molecules26175403 (PMC8434405; doi:10.3390/molecules26175403)
Supplement: Supplementary file 1 [file molecules-26-05403-s001.zip › molecules-1333871-supplementary.pdf]

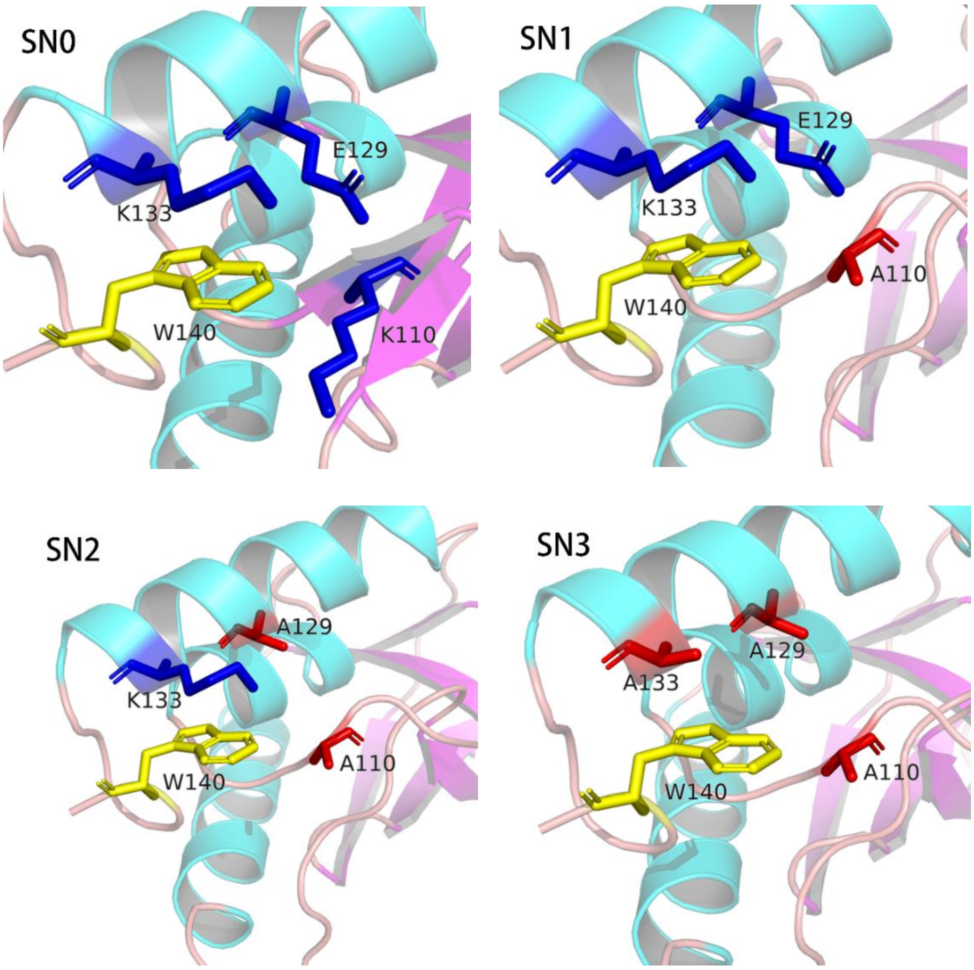

Figure S1. The local structure of the mutation system named SN0, SN1, SN2, SN3. The non-mutated ones are shown in blue, the mutated ones are shown in red. W140 are in yellow.

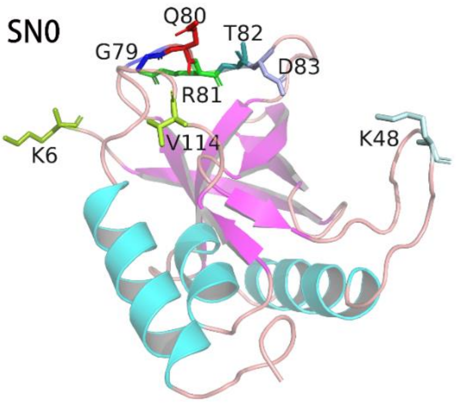

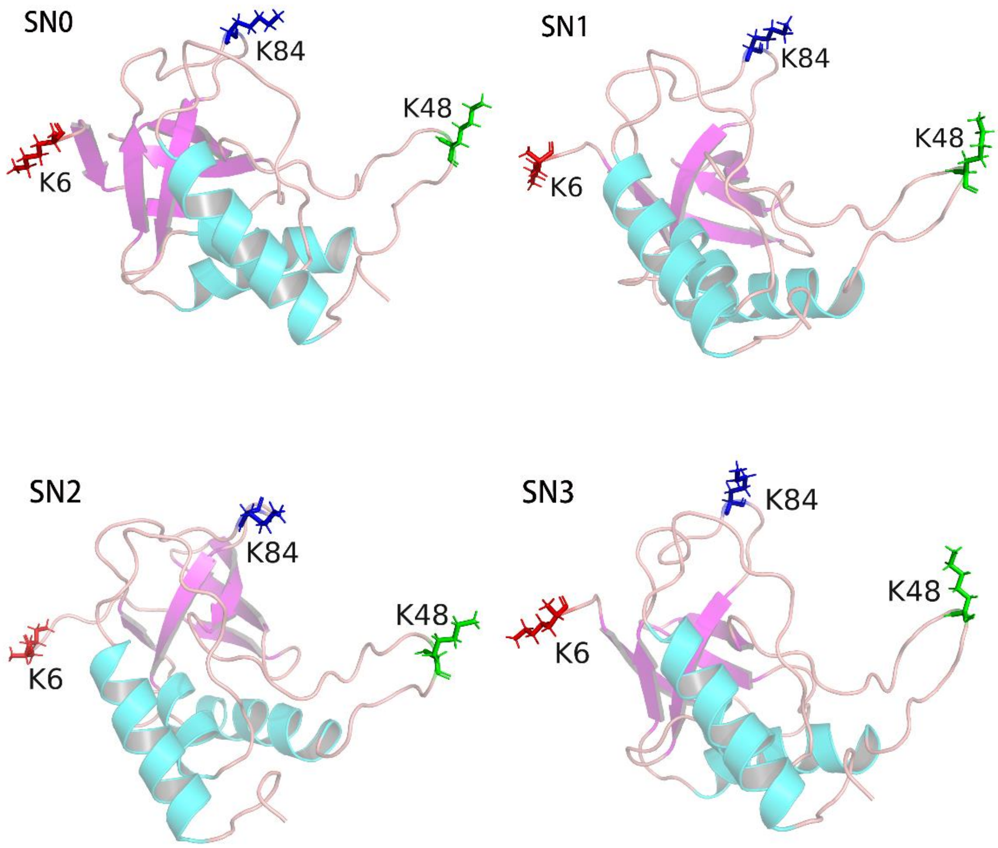

Figure S2. The position of the residues with large changes in RMSF in the protein structure (SN0, SN1, SN2, SN3).

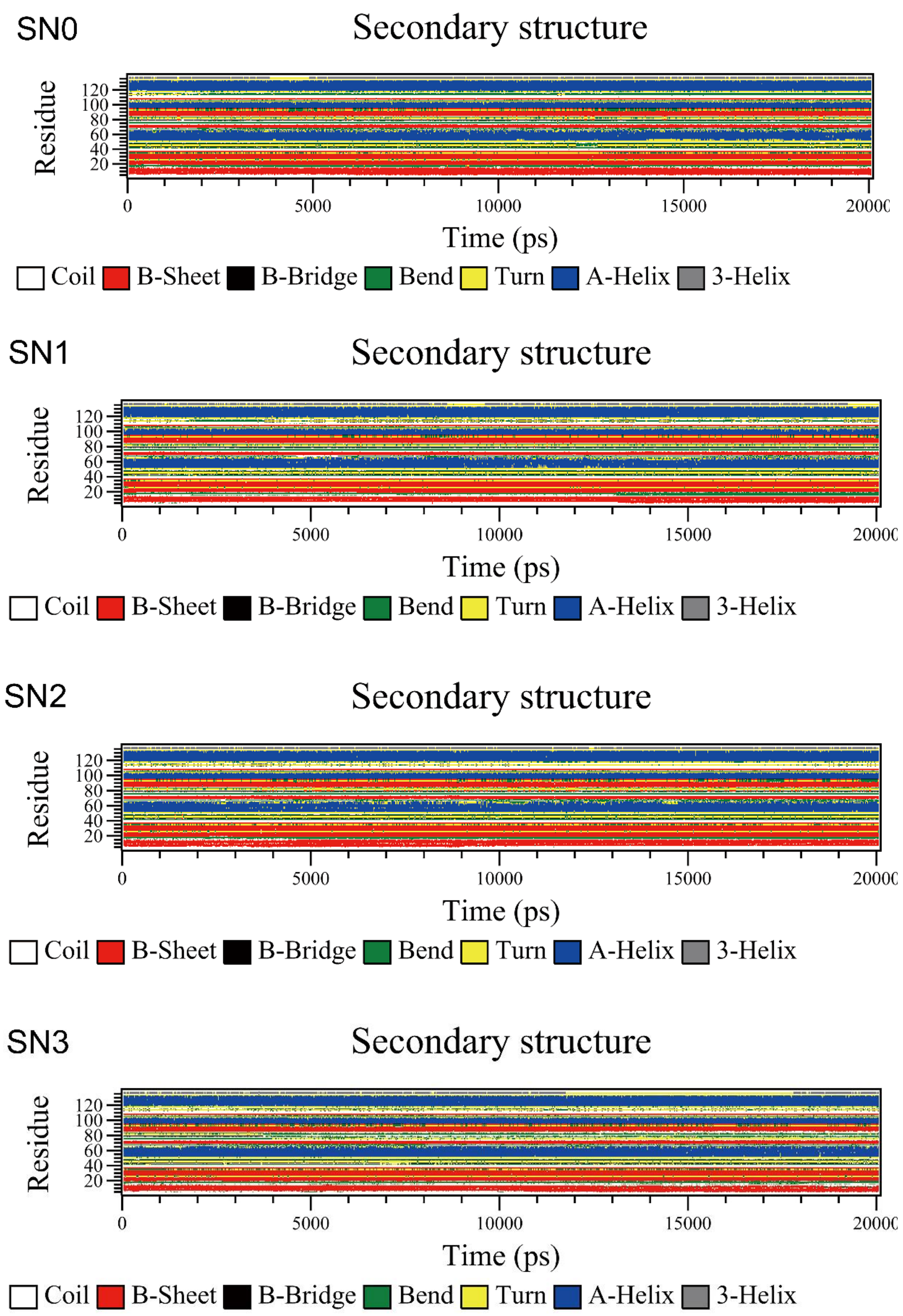

Figure S3. Secondary structure of mutation systems during the 20ns.

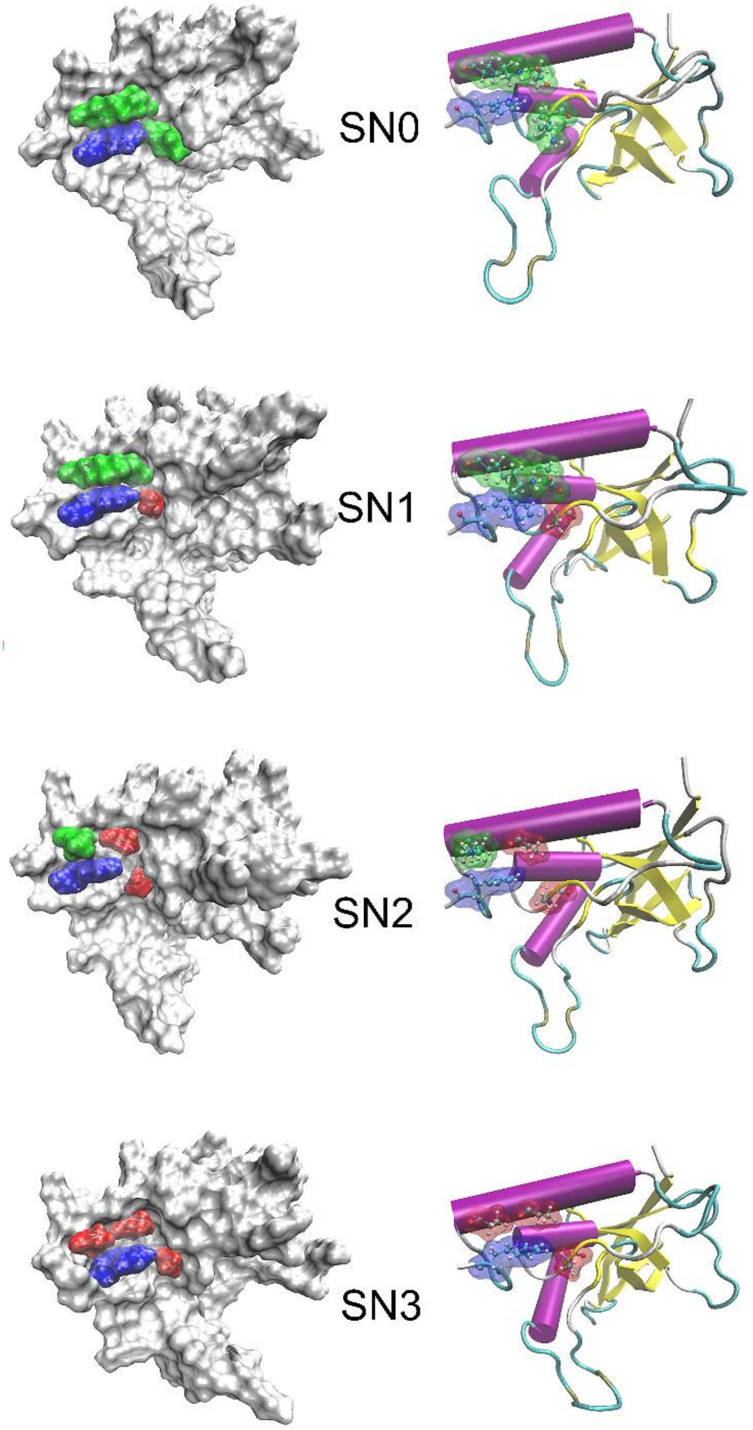

Figure S4. Structural comparison of mutant systems.

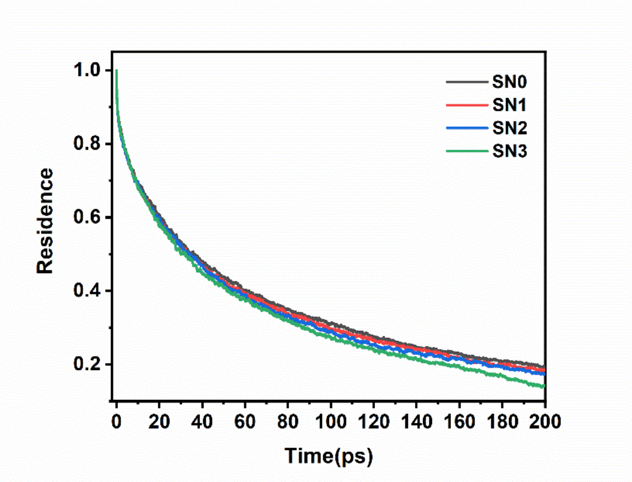

Figure S5. The residence dynamics of hydration water in mutation systems.

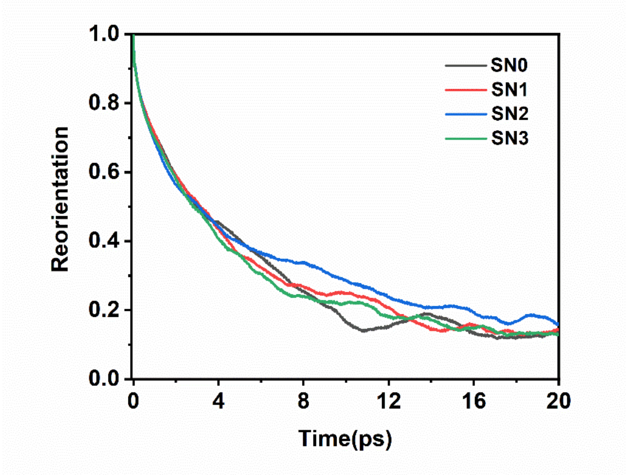

Figure S6. The reorientation dynamics of hydration water in mutation systems.
